# Analysis of biomass productivity and physiology of *Nitrososphaera viennensis* grown in continuous culture

**DOI:** 10.3389/fmicb.2023.1076342

**Published:** 2023-02-16

**Authors:** Michael Melcher, Logan H. Hodgskiss, Mohammad Anas Mardini, Christa Schleper, Simon K.-M. R. Rittmann

**Affiliations:** ^1^Archaea Biology and Ecogenomics Division, Department of Functional and Evolutionary Ecology, University of Vienna, Vienna, Austria; ^2^Arkeon GmbH, Tulln a.d. Donau, Austria; ^3^Archaea Physiology & Biotechnology Group, Department of Functional and Evolutionary Ecology, University of Vienna, Vienna, Austria

**Keywords:** archaea, bioreactor, closed batch, batch, fed-batch, chemostat, carbon dioxide, biofilm

## Abstract

Microbial ammonia oxidation is the first and usually rate limiting step in nitrification and is therefore an important step in the global nitrogen cycle. Ammonia-oxidizing archaea (AOA) play an important role in nitrification. Here, we report a comprehensive analysis of biomass productivity and the physiological response of *Nitrososphaera viennensis* to different ammonium and carbon dioxide (CO_2_) concentrations aiming to understand the interplay between ammonia oxidation and CO_2_ fixation of *N. viennensis*. The experiments were performed in closed batch in serum bottles as well as in batch, fed-batch, and continuous culture in bioreactors. A reduced specific growth rate (μ) of *N. viennensis* was observed in batch systems in bioreactors. By increasing CO_2_ gassing μ could be increased to rates comparable to that of closed batch systems. Furthermore, at a high dilution rate (*D*) in continuous culture (≥ 0.7 of μ_max_) the biomass to ammonium yield (Y_(X/NH3)_) increased up to 81.7% compared to batch cultures. In continuous culture, biofilm formation at higher *D* prevented the determination of *D*_crit_. Due to changes in Y_(X/NH3)_ and due to biofilm, nitrite concentration becomes an unreliable proxy for the cell number in continuous cultures at *D* towards μ_max_. Furthermore, the obscure nature of the archaeal ammonia oxidation prevents an interpretation in the context of Monod kinetics and thus the determination of *K*_S_. Our findings indicate that the physiological response of *N. viennensis* might be regulated with different enzymatic make-ups, according to the ammonium catalysis rate. We reveal novel insights into the physiology of *N. viennensis* that are important for biomass production and the biomass yield of AOA. Moreover, our study has implications to the field of archaea biology and microbial ecology by showing that bioprocess technology and quantitative analysis can be applied to decipher environmental factors affecting the physiology and productivity of AOA.

## Introduction

Nitrification is the oxidation of ammonia (NH_3_) to nitrate (NO_3_^−^) *via* the intermediate nitrite (NO_2_^−^). Ammonia oxidation is the first and usually rate limiting step in nitrification and is therefore important for the global nitrogen cycle. For over a century ammonia oxidation was thought to be performed by the few bacterial genera *Nitrosomonas*, *Nitrosococcus*, and *Nitrosospira,* until 20 years ago when evidence started to accumulate that archaea might play an important role in this process as well (Venter et al., 2004; Könneke et al., 2005; Treusch et al., 2005). *Nitrosopumilus maritimus* was the first isolate from a marine aquarium (Könneke et al., 2005; Qin et al., 2017) followed by *Nitrososphaera viennensis* (Tourna et al., 2011; Stieglmeier et al., 2014) from garden soil. Since then multiple isolates and enrichments of ammonia oxidizing archaea (AOA) have been established and characterized to further improve our understanding of these ubiquitously abundant organisms (De La Torre et al., 2008; Blainey et al., 2011; Lehtovirta-Morley et al., 2011, 2016; Lebedeva et al., 2013; Jung et al., 2014; Zhalnina et al., 2014; Santoro et al., 2015; Qin et al., 2017; Sauder et al., 2017, 2018; Abby et al., 2018; Daebeler et al., 2018; Alves et al., 2019; Bayer et al., 2019; Nakagawa et al., 2021). Viable habitats include oceanic crust (Nunoura et al., 2013; Jørgensen and Zhao, 2016; Zhao et al., 2020), deep sea sediments (Francis et al., 2005; Park et al., 2008; Nunoura et al., 2018; Vuillemin et al., 2019; Zhao et al., 2019; Kerou et al., 2021), marine water column (Qin et al., 2017; Bayer et al., 2019; Santoro, 2019), oxygen minimum zones (Bristow et al., 2016), various kinds of soils (Lehtovirta-Morley et al., 2011, 2016; Tourna et al., 2011; Jung et al., 2014; Zhalnina et al., 2014; Alves et al., 2019), fresh water ecosystems (French et al., 2012, 2021; Sauder et al., 2018), waste water treatment plants (Mußmann et al., 2011; Sauder et al., 2017), terrestrial hot springs (De La Torre et al., 2008; Reigstad et al., 2008; Dodsworth et al., 2011; Abby et al., 2018; Daebeler et al., 2018; Luo et al., 2020), and human skin (Probst et al., 2013; Moissl-Eichinger et al., 2017). AOA outnumber their bacterial counterparts, ammonia oxidizing bacteria (AOB), by orders of magnitude in many habitats (Karner et al., 2001; Leininger et al., 2006; Adair and Schwartz, 2008; Nicol et al., 2008; Hollibaugh et al., 2011), but their contribution to the nitrification process is still not completely resolved. While all known AOA and AOB are confined to oxidize NH_3_ to NO_2_^−^, another group of bacteria was recently identified which are able to perform the complete oxidation of NH_3_ to NO_3_^−^, thus given the name Comammox (Daims et al., 2015; van Kessel et al., 2015).

In bacteria, NH_3_ is oxidized to hydroxylamine (NH_2_OH) by the enzyme ammonia monooxygenase (AMO) (Hollocher et al., 1981; Hyman and Wood, 1985) which is then further oxidized to nitric oxide (NO) by the hydroxylamine oxidoreductase (HAO) (Hooper and Terry, 1979; Caranto and Lancaster, 2017). The enzyme responsible for the oxidation of NO to NO_2_^−^ is still unknown. Unlike the bacterial NH_3_ oxidation pathway, the archaeal one is still very poorly characterized. Only the oxidation of NH_3_ to NH_2_OH is inferred to be catalyzed by an AMO (Vajrala et al., 2013) which is very distantly related to bacterial AMO and all other enzymes of the copper membrane monooxygenase superfamily (CuMMO) (Alves et al., 2018). However, a counterpart to the bacterial HAO is still missing in archaea. Two models are currently proposed, one that mimics the bacterial pathway (Lancaster et al., 2018) and another one where ammonia is oxidized to hydroxylamine which would then be further oxidized to NO_2_^−^ with NO as a co-substrate (Kozlowski et al., 2016). NO would be produced by the reduction of NO_2_^−^ by a proposed nitrite reductase (NirK), which is highly expressed in most AOA, but whose role is still ambiguous as AOA do not perform nitrifier denitrification, unlike AOB (Wrage-Mönnig et al., 2018).

One important factor in niche differentiation of organisms is their substrate affinity, which is described either as reaction rate (*v*) based on the *K_m_* value (Eq. 1) or as specific growth rate (μ) based on the *K_S_* value (Eq. 2). In a steady state the residual substrate concentration (*S*) remains constant over time.


Eq. 1 $$v=\frac{v_{\text{max}}\cdot \left[S\right]}{K_{m}+\left[S\right]}$$

Eq. 2 $$\mu =\frac{\mu_{\text{max}}\cdot \left[S\right]}{K_{S}+\left[S\right]}$$

AOA are notoriously difficult to grow and produce only very little biomass and therefore most information about AOA is provided in the form of the apparent substrate affinity (*K*_*m*(app)_), which is based on whole cell activity measurements of molecular oxygen (O_2_) consumption in micro-respiratory chambers (Kits et al., 2017; Jung et al., 2022). Most AOA are considered oligotrophs and their *K*_*m*(app)_ values range from 0.1–1 μmol L^−1^ NH_3_ and NH_4_^+^ for marine strains, 1–10 μmol L^−1^ for soil or thermophilic strains to 0.1–10 mmol L^−1^ for the soil clade Nitrosocosmicus. Comammox have *K*_*m*(app)_ in the range of 0.1–10 μmol L^−1^ NH_3_ and NH_4_^+^ and are thus considered also oligotrophs while AOB are rather considered eutrophs with *K*_*m*(app)_ between 0.1 and 100 mmol L^−1^ NH_3_ and NH_4_^+^ (Jung et al., 2022). In an attempt to measure the *K_S_* value of a fresh water AOA enrichment in batch cultures, no effect of the initial substrate concentration on μ was observed, suggesting that the *K_S_* is much lower than the lowest tested concentration of about 15 μmol L^−1^ NH_4_^+^ (French et al., 2021). In a chemostat experiment, *N. maritimus* was grown with 150 μmol L^−1^ NH_4_^+^ and different dilution rates (*D*) of 0.010, 0.023 and 0.032 h^−1^ (μ_max_ is 0.036 h^−1^) to investigate the influence of μ on the lipid composition of the organism. NO_2_^−^ concentration only varied by a maximum of 7% (Hurley et al., 2016) but no information was given about the residual substrate concentration.

*Nitrososphaera viennensis* was isolated from garden soil and grows optimal at 42°C (Stieglmeier et al., 2014) with the addition of pyruvate to scavenge reactive oxygen species (ROS) that are endogenously produced (Kim et al., 2016). Cell concentrations and μ are usually approximated by NO_2_^−^ concentrations, as they have been shown to correlate well (Tourna et al., 2011; Stieglmeier et al., 2014). However, *N. viennensis* produces far too little biomass to measure optical density or dry cell weight.

As the different forms of cultivation systems are rarely discussed in the field of nitrification (except for waste water treatment plants), a short overview of the most commonly used systems shall be given here. In general, systems can be distinguished by the level of which they allow energy and matter to be transferred through them. Closed systems (transfer of energy but not matter) and open systems (transfer of energy and matter) are the extreme cases of reality and the isolated system (no transfer of energy or matter) serves as an important theoretical construct that can only be asymptotically approached by closed systems. Cultivation systems are characterized by the level of transfer of matter and in analogy, closed batch (e.g., serum flask with rubber stopper) is a closed system (Taubner and Rittmann, 2016; Mauerhofer et al., 2019), continuous culture (e.g., bioreactor with gassing and in- and outflow of medium) is an open system, with the openness of the system depending on the transfer rates. Open batch (e.g., Erlenmeyer flask with cotton plug, bioreactor with gassing) and fed-batch (e.g., bioreactor with gassing and inflow of medium) are open system intermediates in between the two extremes (Mauerhofer et al., 2019).

Batch systems are probably the most common cultivation systems used in microbiology, because they are very easy to set up and require little technological infrastructure. Closed batch is usually only used if an atmosphere different from air is required (Mauerhofer et al., 2019; Hanišáková et al., 2022). A major disadvantage of batch systems are the changing substrate concentrations that lead to a very heterogeneous biomass which can complicate analysis. Fed-batch systems consist of a shorter batch phase followed by a feed phase, where usually a concentrated feed medium is used to increase the biomass concentration but at the same time avoid substrate inhibition. By using an exponential feeding strategy μ can be kept constant and a relatively homogeneous biomass can be produced, as long as no product inhibition or other limitations hamper growth. In continuous culture systems a stable flow of medium is maintained after an initial batch phase and by changing the dilution rate (*D*, Eq. 3), different steady states can be established by changing the flow rate (*Q*) or the volume of the culture (*V*).


Eq. 3 $$D=\frac{Q}{V}$$

A system is usually considered to be in a steady state after five volume exchanges (99.3% of medium exchanged) while all parameters are kept constant. As a result, the produced biomass is very homogeneous, because in a steady state μ is equal to *D*. Technically speaking, productivities of organisms that are grown in continuous culture are up to tenfold higher compared to batch systems (Herbert et al., 1956), because the system can be stably operated near μ_max_ and downtime for disassembly, sterilization and reassembly of the bioreactor becomes increasingly negligible with longer operation times. Continuous cultures are also excellent tools to study the physiology of microorganisms due to the high level of control and the extended periods of time a steady state can be maintained. A typical application is the determination of the *K_S_* value by varying *D* and measuring the residual substrate concentration (*S*), thereby relating μ to *S* and thus allowing to calculate *K_S_* when assuming Monod kinetics. However, biofilm formation, genetic adaptation or other factors might flaw the determination of such values with extended process runtimes.

In stirred tank reactors gas is usually supplied by a fumigation tube at the bottom of the reactor. The gas transfer rate, which is often a limiting factor for fast growing organisms, can be increased by, e.g., the gassing rate, stirrer speed and operating pressure (Rittmann et al., 2018; Pappenreiter et al., 2019). Due to the low μ_max_ and biomass concentration (*X*) of AOA the gas transfer rate is not a limiting factor but rather needs to be considered because of the possibility to strip important metabolic intermediates (like NO) from the system.

All cultivated AOA are chemolithoautotrophs and fix CO_2_ by a modified version of the hydroxypropionate/hydroxybutyrate cycle (Könneke et al., 2014). For the cultivation of AOA and AOB in bioreactors CO_2_ is usually supplied by air and NaHCO_3_ that is used to titrate the pH and act as C-source (Hurley et al., 2016; Sedlacek et al., 2020; French et al., 2021). Given the fast reaction kinetics of the aqueous carbonate system, the liquid phase in a gassed reactor will tend to be in equilibrium with the supplied gas mix independently of NaHCO_3_ titration (Wang et al., 2010).

The aim of this study was to investigate the growth behavior of *N. viennensis* in continuous culture systems to develop a biomass production process that would enable biochemical studies of the organism to eventually elucidate the energy metabolism of AOA. Growth conditions were optimized to ensure that only NH_3_ acts as a limiting substrate while characterizing process parameters such as *K_S_*, biomass to substrate yield (Y_(X/NH3)_) and the critical dilution rate (*D*_crit_) at which the organism will be washed out, for the bioprocess development. To further increase the biomass productivity per reactor volume we also established a continuous culture at a higher substrate concentration.

## Materials and methods

### Cultivation of *Nitrososphaera viennensis*

*Nitrososphaera viennensis* was routinely grown as batch cultures in 30 mL Quickstart universal containers (STERILIN) at 42°C in freshwater medium (FWM) (De La Torre et al., 2008; Tourna et al., 2011) amended with non-chelated trace element solution (MTE) (Könneke et al., 2005), 7.5 μmol L^−1^ FeNaEDTA, 2 mmol L^−1^ NH_4_Cl, 2 mmol L^−1^ NaHCO_3_, 1 mmol L^−1^ sodium pyruvate, and 10 mmol L^−1^ HEPES buffer (final pH 7.5). Cultures used as inoculum for bio reactors were inoculated with 1% (v/v), but for regular culture maintenance 1:10^6^ (v/v) inoculum was used. Long term storage works best by aliquoting late exponential cultures without any additives and storing them at −70°C. For reviving the culture 5% (v/v) inoculum should be used and a lag phase of 1–2 days need to be expected. Growth was followed by measuring NO_2_^−^ production and NH_3_ consumption as described before (Tourna et al., 2011).

### Batch

To elucidate the effect of gassing on μ, *N. viennensis* was grown in 2 L all-glass bioreactors (Eppendorf AG, Hamburg, Germany) filled with 1.5 L. The medium composition and conditions were the same as for batch cultures, except for omitting HEPES buffer, and the inoculum was 5% (v/v). The pH was controlled at pH 7.50 (± 0.05 deadband) using 0.5 mol L^−1^ HCl and 0.5 mol L^−1^ NaOH for titration. Dissolved molecular oxygen concentration (dO_2_) was measured with optodes (Hamilton) and recorded but not controlled. Cultures were continuously stirred at a rate of 400 rpm and depending on the experiment not gassed (in- and off-gas clamped off), gassed with 2 sL L^−1^ h^−1^ air, gassed with 1 sL L^−1^ h^−1^ air or 1 sL L^−1^ h^−1^ air/N_2_ mix (12.6 Vol.-% O_2_). In the last batch experiment cultures were gassed with 2 sL L^−1^ h^−1^ air for 63 h, in- and off-gas clamped off and NO-donor 2,2′-(2-Hydroxy-2-nitrosohydrazinylidene)bis-ethanamine (DETA NONOate) added *via* a septum in different concentrations (0.15, 0.6 and 2.4 μmol L^−1^, sterile H_2_O as control). After 25 h one reactor was gassed with 2 sL L^−1^ h^−1^ air, one with 2 sL L^−1^ h^−1^ 99% air 1% CO_2_ mixture, one was kept clamped off and one clamped off and 2 mmol L^−1^ NaHCO_3_ were added *via* a septum.

### Chemostat

Chemostat cultures were grown like batch cultures but without NaHCO_3_ in the medium, instead the cultures were gassed with an air/CO_2_ mix of 0.5, 1 or 2 Vol.-% CO_2_ with 2 sL L^−1^ h^−1^ gassing rate. The culture volume was 1.5 L but had to be reduced to 1.1 L at the highest *D* of 0.07 h^−1^ to not exceed the maximum pump rate of 100 mL h^−1^. The chemostats were started in late exponential growth phase of the batch and due to the relatively low μ of the organism, the minimum volume exchanges for steady states, after every applied or observed change, were reduced from the usual five (99.3% (v/v) exchanged) to three exchanges (95.0% (v/v) exchanged) to speed up the experiment.

Peristaltic pumps were calibrated by weighing feed- and harvest bottles every day to avoid drifting over time and the reactor volume was corrected with every sampling. Deviations of the reactor volume were usually between 1 and 3%. Outliers of BR1 and BR2 at 1,271 h and 1,295 h were caused by technical issues of the outflow pumps after the reduction of the working volume to 1.1 L, causing a culture volume increase to about 2 L over night and thus a decrease in residual NH_4_^+^. Outliers of BR2 at 1,343 h and 1,346 h were due to a ripped inflow pump tube, causing a temporary reduction of culture volume to about 0.9 L, a decrease in residual NH_4_^+^ and a contamination with small rod-shaped microorganisms that was treated with kanamycin.

### Washing experiment

To estimate the activity of the biofilm on the reactor walls, the planktonic cells were removed by draining the reactors and refilling them with fresh and sterile medium twice. At a working volume of 1.5 L and 7.5 mL remaining in the bioreactors, a dilution of the planktonic culture of 1:40,000 was achieved (43 nmol L^−1^ NO_2_^−^ remaining). After that the continuous cultures were started again with a *D* of 0.065 h^−1^. During the washing procedure, precipitated carbonate detached from the acid/base/medium inlet in BR2 and started to scrape off the biofilm from the reactor wall, thereby creating a constant loss of biofilm to the system.

### Fed-batch

The fed-batch experiment started with two batch cultures of 1.1 L gassed with an air/0.5 Vol.-% CO_2_ mix of 2 sL L^−1^ h^−1^. The 400 mL feed medium contained 32 mmol L^−1^ NH_4_Cl (10 mmol L^−1^ final), 4.75 mmol L^−1^ pyruvate (2 mmol L^−1^ final), 4.75x MTE (2x final), 4.75x FeNaEDTA (2x final) and was exponentially fed in the beginning to maintain a μ of 0.02 h^−1^. Due to unreliable pump rates the feed mode was changed to linear with a feed rate of 2 mL h^−1^. The pyruvate concentration in the feed medium for the continuous culture was increased to 5 mmol L^−1^ to avoid pyruvate depletion which resulted in stagnating cell concentration during the fed-batch.

### Sampling

Samples were taken from the bioreactors after the first 2 mL were discarded to remove old culture from the tubing. One micro liter aliquots were pipetted into 1.5 mL reaction tubes and centrifuged at 4°C for 30 min at 23,000 g in a benchtop centrifuge (Eppendorf Centrifuge 5424 R). Supernatant was removed and used directly for NO_2_^−^ and NH_3_ measurements while the cell pellets were stored at −20°C for DNA extraction.

### Exchanging feed and harvest bottles

Basic FWM was autoclaved in 5 L Schott bottles and supplements were added in a laminar flow hood after room temperature was reached. The GL45 caps with tubing and 0.2 μm filter were always transferred from one bottle to the next, while keeping the inlet and outlet tubes clamped off. After attaching the bottles back to the reactor tubes *via* luer locks, the connections were put into beakers with boiling water for at least 10 min to reduce the contamination risk, before opening the clamps and restarting the pumps. While BR2 was slightly contaminated due to ripped feed pump tubing, BR1 remained clean over the whole run (103 days with 36 bottle exchanges). The contamination of BR2 occurred at 1,343 h and lasted at most up to 2,041 h.

### DNA-extraction

DNA from cell pellets was extracted by bead beating using SDS-buffer, phenol/chloroform/isoamylalcohol [25:24:1 (v/v/v), Fisher BioReagents] and chloroform/isoamylalcohol [24:1 (v/v)] as already described elsewhere (Zhou et al., 1996; Griffiths et al., 2000; Rittmann and Holubar, 2014; Abby et al., 2018).

### PCR

DNA samples were checked for bacterial contamination using the 16S rRNA primers Eubac27F 5′-AGA GTT TGA TCC TGG CTC AG-3′ and Eubac1492R 5′-GGT TAC CTT GTT ACG ACT T-3′. PCR conditions were 95°C for 5 min as initialization, followed by 35 cycles of 30 s denaturing at 94°C, 30 s primer annealing at 55°C, 2 min elongation at 72°C, and finishing with final elongation at 72°C for 10 min. Reactions were done in 25 μL containing 2 μL sample, 0.15 μL GoTaq Polymerase, 5 μL 5x Flexi Buffer, 2 μL MgCl_2_ 25 mmol L^−1^, 0.5 μL dNTP 10 mmol L^−1^, 0.25 μL BSA 20 mg mL^−1^, 1.25 μL Primer F 10 μmol L^−1^, 1.25 μL Primer R 10 μmol L^−1^, 12.6 μL nuclease free water.

### Quantitative PCR

The archaeal 16S rRNA gene was quantified to estimate the cell number using the primers Arch931F 5′-AGG AAT TGG CGG GGG AGC A-3′ (Jackson et al., 2001) and Arch1100R 5‘-BGG GTC TCG CTC GTT RCC-3′ (Øvreås et al., 1997) in triplicate 20 μL reactions containing 10 μL qPCR Master Mix 2x (Luna Universal qPCR Master Mix, NEW ENGLAND BioLabs Inc.), 1 μmol L^−1^ of each primer, nuclease free water and 4 μL DNA sample (diluted to have approximately 10 ng per reaction) or standard. Reactions were performed in a qPCR (BIO-RAD CFX Connect Real-Time System) with the following conditions: initialization at 95°C for 2 min, 40 cycles of 30 s denaturing at 95°C, 30 s joint annealing-extension at 60°C, and extension with fluorescence measurement at 60°C for 30 s. The specificity of qPCR products was confirmed by melting curve analysis. Standards were prepared by amplifying the 16S rRNA gene of *N. viennensis* under the conditions as described above in a 50 μL reaction using 2 μL DNA template and the archaeal 16S rRNA primers A109F 5‘-ACK GCT CAG TAA CAC GT-3′ (Großkopf et al., 1998) and A1492R GYY ACC TTG TTA CGA CTT-3′ (Nicol et al., 2008). PCR product was cleaned up using the Machery-Nagel DNA cleanup kit and DNA concentration was measured with Qubit™ dsDNA BR Assay Kit (Thermo Fisher Scientific) before preparing serial dilutions with nuclease free water. The standard curve had an efficiency of 93.38% and an R^2^ of 0.999.

### Extrapolating cell number and biomass from DNA concentration

DNA concentration of extracted samples was measured with Qubit™ dsDNA HS Assay Kit (Thermo Fisher Scientific) and several samples with concentrations ranging from 3.58 to 35.5 ng μL^−1^ were quantified by qPCR as described above. Using a linear regression model the mass of DNA was correlated to the cell number, assuming that each cell contained a single copy of the 16S rRNA gene. Using this model one copy of 16S rRNA correlates to 3.42 fg which is 25.6% more than the calculated mass of a *N. viennensis* genome of 2.72 fg (2.52 Mbp genome size). Assuming a cell diameter of 0.75 μm (0.6–0.9 μm cell diameter) and a density of 1.1 g cm^−3^ a theoretical *N. viennensis* cell would weight 2.43·10^−13^ g or 243 fg.

### Harvesting of biomass

The outflow of the continuous cultures was collected in sterile 5 L bottles and usually harvested once a week by concentrating the biomass using a tubular centrifuge (CEPA) at 40,000 rpm and subsequent centrifugation of the cell concentrate in a bucket centrifuge (Thermo SCIENTIFIC Sorvall LYNX 4000 Centrifuge) at 4°C, 24,470 g for 40 min. The pellets were resuspended in a small amount of supernatant and split up into pre-weighted 2 mL reaction tubes which were centrifuged in a benchtop centrifuge (Eppendorf Centrifuge 5424 R) at 4°C for 30 min at 23,000 g. Supernatant was removed, reaction tubes weighed and stored at −70°C.

### Equations

#### Active biofilm estimation

To estimate the amount of biofilm biomass contributing to nitrification, biofilm BR1 was modeled as a continuously stirred tank reactor (CSTR) at the final time point taken. BR1 was chosen based off of its relative stability compared to BR2. The change of substrate with relation to time can be described using the following equation:


Eq. 4 $$V\frac{dS}{dt}=QS_{i}-QS+r_{f}A_{f}L_{f}+r_{S}V$$

Where *V* is the volume of the reactor, *Q* is the flow rate, *S_i_* is the concentration of ammonium in the inflow, *S* is the concentration of ammonium in the outflow, *r_s_* represents the reaction of ammonium to nitrite by the planktonic cells, *r_f_* represents the reaction of ammonium to nitrite by biofilm cells, *A_f_* is the surface area of the reactor covered by biofilm, and *L_f_* is the height of the biofilm. The volume of the biofilm is assumed to be negligible when compared to the planktonic volume (and therefore total volume) of the reactor system.

The reaction of ammonium to nitrite (mmol L^−1^ h^−1^) can be represented by:


Eq. 5 $$r=\frac{-1}{Y_{XS}}\mu X$$

Where Y_(X/NH3)_ represents the yield of *N. viennensis* biomass from ammonia oxidation (mg biomass/mmol ammonium), μ is the specific growth rate (h^−1^), and *X* represents the respective biomass concentration (mg/L; planktonic or biofilm).

Substituting the reaction rate into the substrate mass balance:


Eq. 6 $$V\frac{dS}{dt}=Q\left(S_{i}-S\right)+\left(\frac{-1}{Y_{XS}}\mu_{f}X_{f}A_{f}L_{f}\right)+\left(\frac{-1}{Y_{XS}}\mu XV\right)$$

Assuming steady state (*dS*/*dt* = 0), a *μ* that is the same for both biofilm and planktonic cells (*μ_f_* = *μ*), and the volume of the biofilm to be surface area multiplied by height (*A_f_L_f_* = *V_f_*), Equation 6 simplifies to:


Eq. 7 $$0=Q\left(S_{i}-S\right)+\left(\frac{-1}{Y_{XS}}\mu X_{f}V_{f}\right)+\left(\frac{-1}{Y_{XS}}\mu XV\right)$$

The exact volume of the biofilm is unknown due to the unknown height. Assuming a homogenous biofilm and equilibrium between the biofilm and planktonic biomass, the total amount of biomass (mg) contributing to nitrification from the biofilm can be determined by solving Equation 7 for *X_f_V_f_*:


Eq. 8 $$X_{f}V_{f}=\frac{-Y_{XS}}{\mu }Q\left(S-S_{i}\right)-XV$$

The right side of the equation is now represented by known variables when using BR1: Y_(X/NH3)_ = 15 mg biomass/mmol ammonium (calculated from batch cultures (assumption for calculations)); μ = 0.048 h^−1^; Q = 0.0975 L h^−1^ (set parameter); *S_i_* = 2 mmol ammonium/L (set parameter); *S* = 0.3295 mmol ammonium/L (measured in effluent); *X* = 13.3 mg biomass/L (measured in effluent); *V* = 1.5 L (set parameter).

## Results

### Effect of gassing rate and in-gas flow composition in batch and closed batch experiments

**Figure 1:**
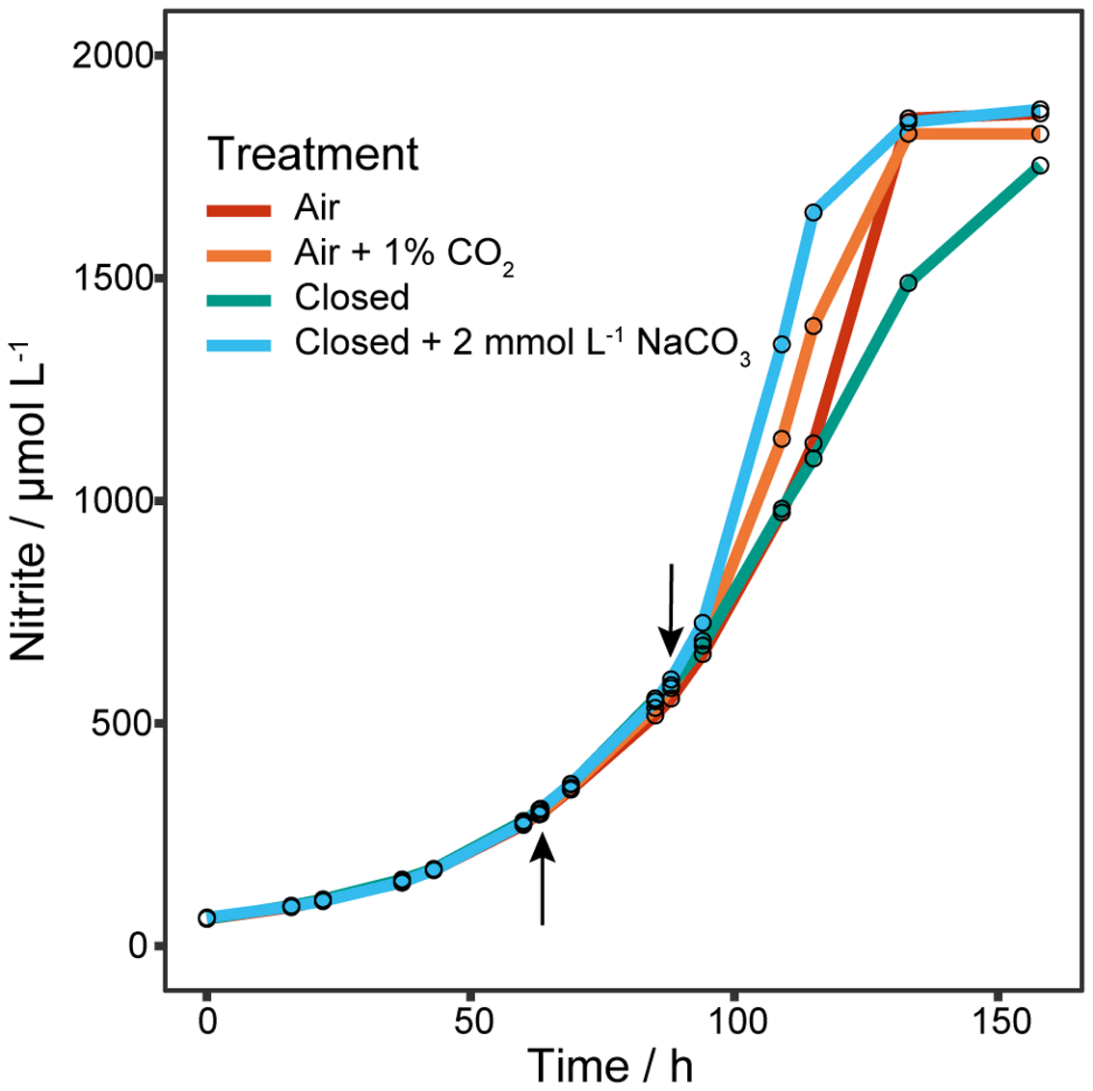
Effect of NO-donor and CO_2_ on μ. Four 1.5 L batch cultures grown in 2 L bioreactors gassed with 2 sL L^−1^ h^−1^ air (*n* = 1). The upward arrow indicates the addition of different amounts of the NO-donor 2,2′-(2-Hydroxy-2-nitrosohydrazinylidene)bis-ethanamine (DETA NONOate) to early exponential cultures, after which gassing was stopped and reactors clamped off to prevent gas exchange. As no effect on the nitrite production was observed, cultures were tested for CO_2_ limitation indicated by the downward arrow. One culture was gassed again with air, one with air plus 1% CO_2_, one was kept closed and one was kept closed but with the addition of 2 mmol L^−1^ NaHCO_3_. Addition of CO_2_ to the in-gas or NaHCO_3_ to the culture medium increased μ, while gassing with air or keeping the reactor closed did not change or reduce μ, respectively.

Initial experiments of *N. viennensis* grown as batch cultures in 2 L bioreactors showed that gassing with air had a detrimental effect on μ even at low gassing rates such as 2 sL L^−1^ h^−1^ (0.0276 h^−1^) when compared to cultures without gassing (0.0445 h^−1^, Supplementary Figure S1). In non-gassed cultures dO_2_ decreased to about 50% of its initial concentration. Therefore, growth under decreased gassing rate and reduced dO_2_ was tested. As shown in Supplementary Figure S2 reducing the gassing rate to 1 sL L^−1^ h^−1^ did not increase μ (0.0267 h^−1^) and reducing dO_2_ to about 60% did decrease μ to 0.0205 h^−1^, which contradicted the results of the first experiment. Due to the hypothesized importance of NO for the archaeal ammonia oxidation pathway (Kozlowski et al., 2016) we decided to test if the reduction of μ is a result of stripping NO from the system by gassing. As shown in Figure 1, addition of the NO-donor 2,2′-(2-Hydroxy-2-nitrosohydrazinylidene)bis-ethanamine (DETA NONOate) to early exponential phase cultures (300 μmol L^−1^ NO_2_^−^ produced) did not show any effect on recovering μ. However, adding 1 Vol.-% CO_2_ to the in-gas or 2 mmol L^−1^ NaHCO_3_ to the culture medium, and keeping the bioreactor closed did increase μ from 0.0259 ± 0.0002 h^−1^ to 0.0338 h^−1^ and 0.0395 h^−1^, respectively. Gassing with air or keeping the reactor closed did not change μ (0.0260 h^−1^) or even reduced it (0.0234 h^−1^), respectively. Hence, the above results showed that the organism was carbon limited likely due to the stripping of CO_2_ from the supplied gassing.

To determine μ_max_ in closed batch systems, *N. viennensis* was grown in serum flasks with 0.5 Vol.-% CO_2_ and different dO_2_ concentrations (Supplementary Figure S3). A slight increase of μ was observed with decreasing dO_2_ from 0.0484 ± 0.0004 h^−1^ to 0.0508 ± 0.0004 h^−1^at 21 Vol.-% to 5 Vol.-% O_2_, respectively (224.7 to 53.5 μmol L^−1^ dO_2_ considering a pressure of 1,106 hPa and an incubation temperature of 42°C). Due to the minor effect of dO_2_ on μ, 21 Vol.-% O_2_ was used to gas the following cultures and a μ_max_ of 0.0484 h^−1^ is therefore considered. Moreover, by using an inoculum of 1:10^6^ (v/v), μ was increased from 0.024 h^−1^ (Stieglmeier et al., 2014) to 0.048 h^−1^ (data not shown).

### Continuous culture experiments at high dilution rates

**Figure 2:**
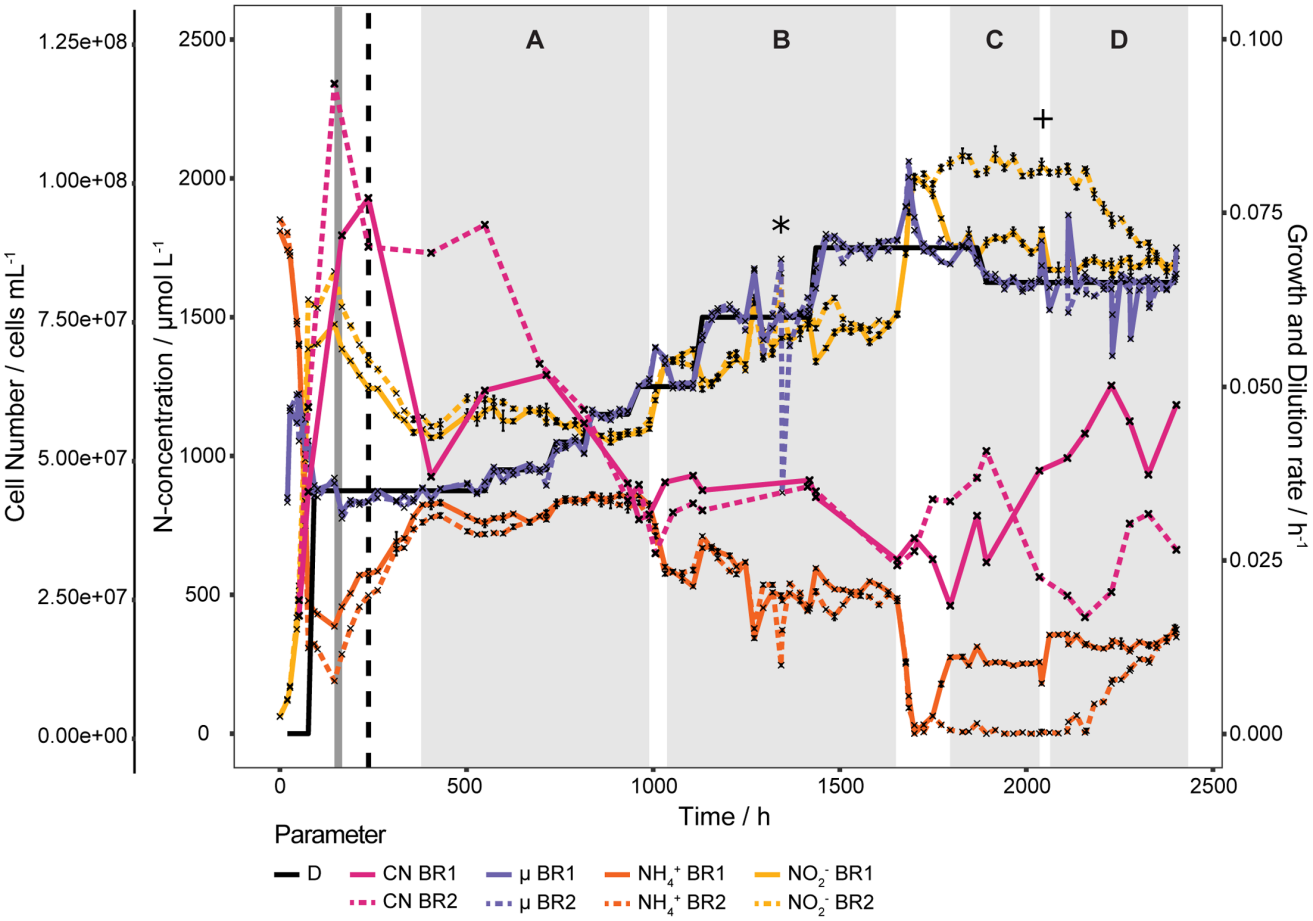
Continuous culture of *N. viennensis* at high *D*. Two continuous cultures with *D* ranging from 0.035 h^−1^ to 0.070 h^−1^ and the development of cell number (CN), NO_2_^−^ derived μ, NH_4_^+^ and NO_2_^−^ concentrations of the cultures grown in the corresponding reactors BR1 and BR2. Cultures were gassed with 1% CO_2_ enriched air at 2 sL L^−1^ h^−1^ gassing rate, except for the period marked in dark grey where the gassing rate was temporarily increased to 10 sL L^−1^ h^−1^. The black dashed line indicates the increase to 2% CO_2_ in the in-gas. Sections A to D signify regions where NH_4_^+^ concentrations stabilized, even though *D* was increased. Spontaneous increases of activity happened between section A and B and section B and C without external influences. Increase of NH_4_^+^ of BR1 from section C to D was due to an increase of reactor working volume and the increase of NH_4_^+^ of BR2 in section D was probably induced by taking larger sampling volumes. The asterisk indicates the point of contamination of BR2 due to a ripped pump tube and the plus sign indicates the point at which the contamination was no longer detectable by PCR of the bacterial 16S rRNA gene. NH_4_^+^ and NO_2_^−^ curves show mean values of technical triplicates and error bars represent the standard deviation of the mean. Points with small standard deviation may not have visivle error bars.

To determine the *K_S_* and *D_crit_* of *N. viennensis* eight different *D* (0.035, 0.038, 0.042, 0.046, 0.050, 0.060, 0.065, and 0.070 h^−1^) were applied in two bioreactors. As shown in Figure 2, at *D* 0.035 h^−1^ *S* stabilized at an unexpectedly high level around 750 μmol L^−1^ NH_4_^+^ and increasing *D* further only had a marginal effect on *S*. In section A (*D* 0.035 to 0.050 h^−1^, 382 to 990 h) *S* stabilized at 801.9 ± 43.2 μmol L^−1^ NH_4_^+^. Activity of both cultures spontaneously increased 58 h after *D* was set to 0.050 h^−1^ (94.5% of medium exchanged) and *S* decreased to 575.2 ± 21.2 μmol L^−1^ NH_4_^+^. The increase of *D* to 0.060 h^−1^ was responsible for the abrupt increase of *S* to 690.1 ± 29.9 μmol L^−1^ NH_4_^+^ which gradually decreased, despite negative outliers, to 456.2 ± 7.9 μmol L^−1^ NH_4_^+^. Overall *S* stabilized at 541.6 ± 68.6 μmol L^−1^ NH_4_^+^ in section B (*D* = 0.050 to 0.070 h^−1^, 1,032 to 1,654 h) and negative outliers at 1,271, 1,295, 1,343, and 1,346 h were due to technical issues (see Materials and Methods). Another spontaneous increase of activity occurred in both cultures 11 days after *D* was set to 0.070 h^−1^ (18 working volume exchanges), which first led to a complete consumption of NH_4_^+^ in both cultures but subsequently two different *S* stabilized. As shown in section C of Figure 2 (*D* = 0.070 and 0.065 h^−1^, 1,796 to 2,035 h), *S* of the BR1 culture stabilized at 262.2 ± 19.9 μmol L^−1^ NH_4_^+^ and at 7.4 ± 11.1 μmol L^−1^ NH_4_^+^ for the BR2 culture. From section C to section D (*D* 0.065 h^−1^, 2,062 to 2,402 h) the reactor volume was increased to minimize the effect of sampling larger volumes which caused *S* of BR1 to increase from 258 to 356 μmol L^−1^ NH_4_^+^ (ratio of concentrations matches the ratio of volumes). *S* of BR2 gradually increased from 4.1 μmol L^−1^ NH_4_^+^ to 375.0 μmol L^−1^ NH_4_^+^ probably as a result of the disturbances caused by the repeated sampling process.

The cell concentrations declined gradually with *D* from 7.67·10^7^ ± 2.11·10^7^ cells mL^−1^ at *D* = 0.035 h^−1^ to 4.36·10^7^ ± 2.18·10^6^ cells mL^−1^ at *D* = 0.046 h^−1^ (549 to 932 h). μ calculated from measured cell concentrations as shown in Supplementary Figure S4 gradually increased with increasing *D* from 0.0362 ± 0.0011 h^−1^ at *D* = 0.035 h^−1^ to 0.0437 ± 0.0007 h^−1^ at *D* = 0.046 and after the activity increase at *D* 0.050 h^−1^ μ increased further to 0.0505 ± 0.0003 h^−1^ (1,107 h). Cell concentrations remained surprisingly stable from *D* = 0.050 to 0.060 h^−1^ at 4.13·10^7^ ± 5.30·10^6^ cells mL^−1^ (1,032 to 1,419 h) and declined to 3.08·10^7^ ± 7.06·10^5^ cells mL^−1^ at *D* = 0.070 h^−1^ (1,654 h) before the second activity increase, after which cell concentrations started to alternate in both reactors. Average NH_4_^+^ and cell concentrations of sections A to *D* are shown in Supplementary Figure S5. *K_S_* and *D_crit_* could not be determined due to the unusual growth behavior and biofilm formation, respectively.

#### Ammonia oxidizing activity of biofilm

**Figure 3:**
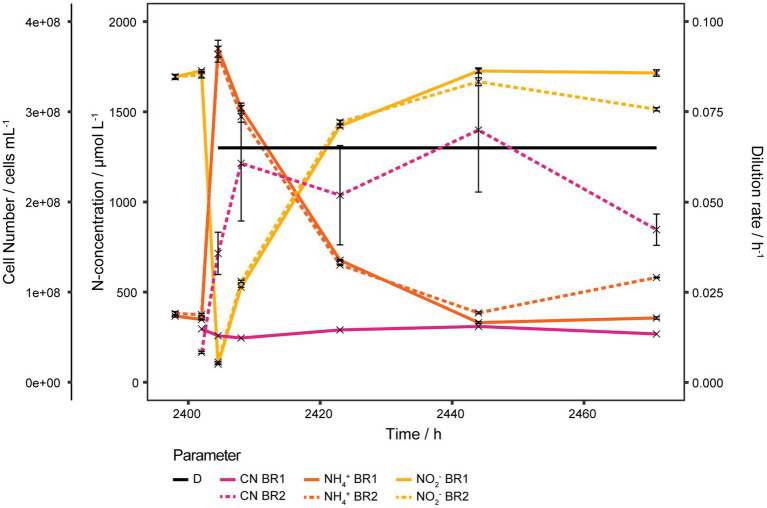
Ammonia oxidizing activity of biofilm. Bioreactors of continuous cultures grown at high *D* were washed, refilled with sterile medium and operated at *D* 0.065 h^−1^ to estimate the activity of the biofilm which had formed on the reactor walls due to the prolonged cultivation time. NH_3_ oxidation started right away and within 2 days the same steady state as before the washing step was recovered in both reactors. Cell number in BR1 was almost unaffected by the washing step, indicating that most planktonic cells were seeded by the biofilm. The steep increase of the cell number in BR2 was due to biomass being scraped off from the reactor wall by precipitate that had formed at the inlet and was washed off during the washing step. Both reactors exhibited very comparable NH_3_ oxidizing activities despite the fact that BR2 lost a considerable amount of its biofilm biomass as it was washed out with the effluent. NH_4_^+^, NO_2_^−^, and cell number of BR2 curves show mean values of technical triplicates and error bars represent the standard deviation of the mean. Points with small standard deviation may not have visible error bars.

Over the course of the experiment a biofilm had formed gradually on the reactor walls. To estimate its contribution to the gross activity, the medium was aseptically removed from the reactors, the reactors were washed once with sterile medium and then refilled with fresh medium to restart the continuous culture at *D* = 0.065 h^−1^. Only a residual NO_2_^−^ concentration of 43 nmol L^−1^ should have been present at the restart of the continuous culture but instead a starting NO_2_^−^ concentration of about 100 μmol L^−1^ was measured in both reactors. As shown in Figure 3 within 2 days the same steady state as before the washing step was recovered in both reactors.

Almost no change in planktonic cell concentration of BR1 was observed after the washing step and the concentration remained stable afterwards between 4.91·10^7^ and 6.19·10^7^ cells mL^−1^. In BR2 the cell concentration increased after the washing step from 3.31·10^7^ ± 1.09·10^6^ to 1.43·10^8^ ± 2.35·10^7^ cells mL^−1^ and increased further to 2.43·10^8^ ± 6.40·10^7^ cells mL^−1^ within the next 3 h as a result of precipitate being washed off of the inlet and subsequently scraping off biofilm from the reactor wall. The cell concentration remained rather stable from there for 36 h up to a concentration of 2.80·10^8^ ± 6.88·10^7^ cells mL^−1^ and decreased to 1.69·10^8^ ± 1.73·10^7^ cells mL^−1^ within the following 27 h.

Thus the biofilm was not only highly active but also contributed considerably to the planktonic cell concentration in the bioreactors by seeding cells. Even though BR2 lost substantial biofilm biomass it still had comparable NH_3_ oxidizing activity to BR1 in which the biofilm was retained.

#### Estimation of active biofilm biomass in BR1

Based on a substrate mass balance and assuming a steady state at time point 2,402 h (Figure 2), 30.9 mg of *N. viennensis* biomass is contributing to nitrification in the biofilm (see Materials and Methods). This represents approximately 1.55 times as much biomass as would be found in the planktonic phase of the reactor. While this number represents the amount of actively nitrifying biomass, it is plausible that the biofilm itself is much more in terms of weight that could be represented by extrapolymer substances (EPS), precipitated bicarbonate, and cells that are potentially inactive due to substrate limitation. While these calculations demonstrate a significant portion (over half) of the nitrification activity is coming from the biofilm, this number could change if it is determined that certain growth parameters, such as the Y_(X/NH3)_ and μ, are differing between the planktonic and biofilm phases. However, within the presented model, the biofilm is affecting the amount of NH_4_^+^ oxidized to NO_2_^−^ and could help explain the NO_2_^−^ productivity within these bioreactors. A large portion of activity from the biofilm is also expected due to NO_2_^−^ being produced even though *D* is higher than the predicted μ_max_. The combined ammonium oxidation of planktonic cells and biofilm, and planktonic cells being seeded from the biofilm, could explain the absence of an expected cellular washout of the reactor.

#### Estimation of biofilm biomass in BR2

An estimate for the total biofilm biomass was obtained from BR2 under the assumption that all biofilm from the bioreactor wall had been scraped off during the biofilm activity experiment. By summing up all cells washed out and subtracting the cells produced during the time of the experiment, the biofilm biomass at the start can be estimated. To assess the amount of cells produced, the total amount of substrate consumed was calculated and then multiplied by the estimated Y_(X/NH3)_. Total cells washed out was calculated as the area under the curve from 2,402 h to 2,471 h (Figure 3) after converting time to volume using the set flow rate. Total ammonia consumed was calculated by subtracting residual ammonia (area under the curve) from total supplied ammonia during the given time span. Using a Y_(X/NH3)_ of 15 mg mmol^−1^ NH_4_^+^ and an estimated mass per cell of 2.3·10^−13^ g cell^−1^ (see Materials and Methods) the amount of cells produced during the time span was calculated to be 167.77 mg. The mass of total cells washed out was calculated to be 378.16 mg. Subtracting produced biomass from total washed out biomass gives an estimate biofilm mass of 210.49 mg. Assuming the amount of biofilm in BR2 from the washout experiment to be representative of the amount of biofilm in BR1 at the end of the chemostat experiment (Figure 2), this data can be combined with the active biofilm estimation (30.9 mg, BR1) calculation to determine the percentage of active biomass in the biofilm. According to these estimations and under the assumption that BR1 and BR2 produced similar amounts of biofilm, only about 14.67% of the cells in the biofilm would have been active.

This percentage should be taken as a conservative estimate as it would be lower if not all biofilm was removed from the reactor during this time period. Biofilm that was observed during takedown or BR2 would indicate this to be a possibility barring the production of new biofilm during the washout experiment.

### Continuous culture experiments at low dilution rates

**Figure 4:**
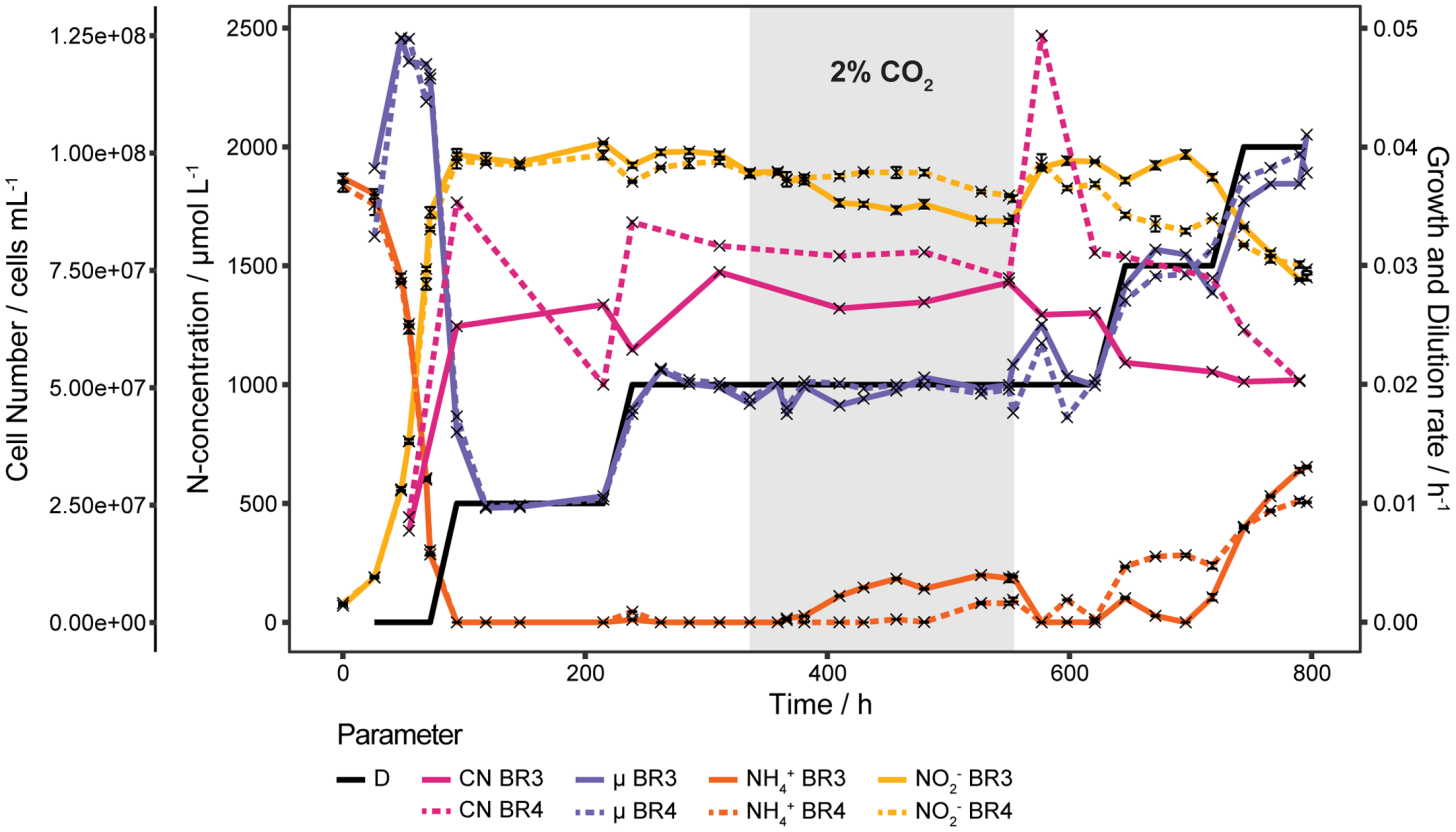
Continuous cultures of *N. viennensis* at low *D*. Two continuous cultures operated at *D* from 0.010 h^−1^ to 0.040 h^−1^ and the development of cell number (CN), NO_2_^−^ derived μ, NH_4_^+^ and NO_2_^−^ concentrations of the cultures grown in the corresponding reactors BR3 and BR4. Cultures were gassed with 2 sL L^−1^ h^−1^ 0.5% CO_2_ enriched air, except for the gray area where CO_2_ was increased to 2%. The elevated CO_2_ concentration induced an increase of NH_4_^+^ with a delay of 55.5 h and 216 h in BR3 and BR4, respectively. NH_4_^+^ was consumed again right after decreasing CO_2_ back to 0.5% which coincided with a temporary increase of the cell number in BR4. NH_4_^+^ and NO_2_^−^ curves show mean values of technical triplicates and error bars represent the standard deviation of the mean. Points with small standard deviation may not have visible error bars.

To get the full picture of how *D* effects *S*, a new continuous culture run was set up with 0.5 Vol.-% CO_2_ enriched air to reduce the precipitation of CO_3_^2−^ both at the inlets and in the medium. Initial *D* was set at 0.01 h^−1^ and all substrate was consumed. As shown in Figure 4 an increase in *D* to 0.020 h^−1^ resulted in a temporary increase of *S* to 11 and 44 μmol L^−1^ for BR3 and BR4 respectively, after which *S* returned to 0 μmol L^−1^. Then CO_2_ was increased to 2 Vol.-% at 96 h after *D* was increased, which surprisingly induced an increase of *S* in BR3 with a 55.5 h delay. *S* in BR3 slowly started to increase from 17 to finally 192 μmol L^−1^ whereas in BR4 *S* concentrations increased up to 95 μmol L^−1^ but only 216 h after the CO_2_ was increased. The reduction of CO_2_ to 0.5 Vol.-% caused an immediate consumption of all NH_4_^+^ in both reactors (553.5 to 577 h). Increasing *D* to 0.030 h^−1^ increased *S* in both reactors, but at this time BR4 showed higher *S* than BR3. A further increase of *D* to 0.040 h^−1^ induced an extended increase of *S* similar to the behavior seen at *D* 0.035 h^−1^.

Cell concentrations remained rather stable over the whole run from *D* of 0.010 h^−1^ to 0.030 h^−1^ at 6.94·10^7^ ± 1.02·10^7^ cells mL^−1^. Only one strong increase from 7.24·10^7^ cells mL^−1^ to 1.23·10^8^ cells mL^−1^ was observed in BR4 directly after decreasing the CO_2_ concentration of the in-gas from 2 Vol.-% to 0.5 Vol.-%. However, the cell concentration decreased again to 7.77·10^7^ cells mL^−1^ before increasing *D* from 0.020 h^−1^ to 0.030 h^−1^ (Figure 4). The amount of CO_2_ in the gas phase seems to affect *S*. Depending on the CO_2_ concentration of 0.5 Vol.-% and 2 Vol.-% in the gas phase *S* starts to accumulate at *D* of 0.030 h^−1^ or 0.020 h^−1^, respectively.

### Continuous cultures at high ammonia concentrations

Before starting a continuous culture with higher substrate concentrations, the inhibitory effect of NO_2_^−^ was determined by batch cultures in 30 mL polystyrene tubes with varying NO_2_^−^ starting concentrations of 0 to 19 mmol L^−1^ and 1 mmol L^−1^ NH_4_^+^. NO_2_^−^ showed only a weak linear inhibitory effect on μ with 0.0342 to 0.0411 h^−1^ for 19 and 0 mmol L^−1^ NO_2_^−^ starting concentrations, respectively (Supplementary Figure S6).

**Figure 5:**
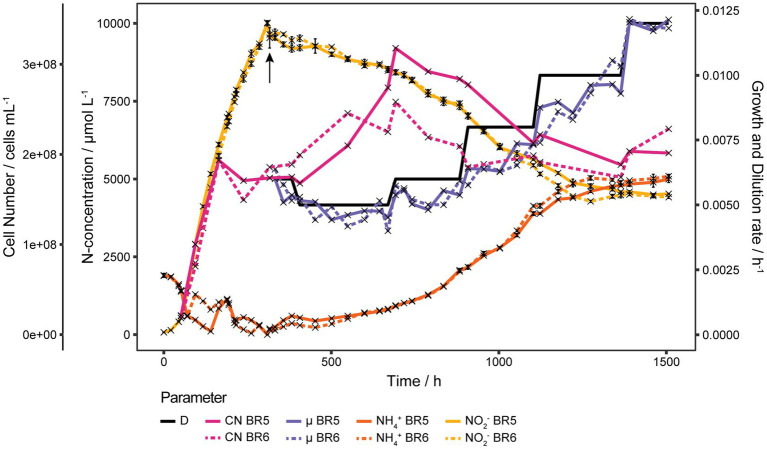
Continuous cultures of *N. viennensis* at high ammonia concentrations. Two continuous cultures gassed with 2 sL L^−1^ h^−1^ 0.5% CO_2_ enriched air, operated at *D* = 0.005 h^−1^ to 0.012 h^−1^ and the development of cell number (CN), NO_2_^−^ derived μ, NH_4_^+^ and NO_2_^−^ concentrations of the cultures grown in the corresponding reactors BR5 and BR6. Batch cultures were grown with 2 mmol L^−1^ NH_4_Cl and 1 mmol L^−1^ pyruvate and then fed with medium containing 32 mmol L^−1^ NH_4_Cl and 4.75 mmol L^−1^ pyruvate to reach a final concentrations of 10 mmol L^−1^ NH_4_Cl and 2 mmol L^−1^ pyruvate. The relative reduction of pyruvate to NH_4_Cl caused a stagnation of the cell number, for which reason pyruvate was added to the cultures at the end of the feed phase (indicated by the arrow) to increase the concentration to 5 mmol L^−1^. Cell number increased as a result of ROS detoxification by pyruvate, but started to decrease again as NH_4_^+^ increased. Once NH_4_^+^ stabilized at *D* = 0.010 h^−1^ increasing *D* further did not result in an increase of NH_4_^+^. NH_4_^+^ and NO_2_^−^ curves show mean values of technical triplicates and error bars represent the standard deviation of the mean. Points with small standard deviation may not have visible error bars.

The bioreactor experiment commenced with a 2 mmol L^−1^ NH_4_^+^ batch, followed by a fed-batch to increase NO_2_^−^ up to 10 mmol L^−1^ and then switching into continuous mode to see the effect of *D* on cell number and *S*. The NH_4_^+^ to pyruvate ratio was changed in the feed from 2 to 5 to avoid excessive pyruvate concentrations that would promote bacterial growth in case of a contamination. For up to 6 mmol L^−1^ NO_2_^−^ the cell number correlated very well with the NO_2_^−^ concentration but then the cell number stagnated in both reactors even though NH_3_ was still oxidized. Pyruvate was added to the reactors at the end of the fed-batch to increase the concentration from 2 mmol L^−1^ to 5 mmol L^−1^ and the feed media for the continuous cultures were also adjusted to 10 mmol L^−1^ NH_4_Cl and 5 mmol L^−1^ pyruvate. Initial *D* was 0.006 h^−1^ to allow the cultures to recover but was further decreased to 0.005 h^−1^ to reduce the increase of *S*. In both cultures *S* appeared to be drifting, which means that μ (based on NO_2_^−^ concentration) was always lower than *D* but still increased with increasing *D* (Figure 5). With such low *D* the residence time would have been far too long to wait for three volume exchanges to establish steady states (25 days at *D* = 0.005 h^−1^). *D* was increased incrementally after at least 200 h from 0.005 to 0.006, 0.008, 0.010 and 0.012 h^−1^. *S* eventually stabilized around 5 mmol L^−1^ at *D* = 0.01 h^−1^ and an increase of *D* to 0.012 h^−1^ did not further increase *S*. The cell number increased in both reactors from 1.76·10^8^ cells mL^−1^ and 1.88·10^8^ cells mL^−1^ at the beginning of the continuous phase up to 3.22·10^8^ cells mL^−1^ and 2.62·10^8^ cells mL^−1^ for BR5 and BR6, respectively, after *D* was increased to 0.06 h^−1^ and from there gradually decreased to about 2.00·10^8^ at *D* = 0.01 h^−1^ (Figure 5).

Even though very low *D* were used in this continuous culture experiments, surprisingly high NH_4_^+^ concentrations were measured. Once *S* stabilized, increases of *D* did not result in an increase of *S* as observed before with 2 mmol L^−1^ cultures. The NH_4_Cl to pyruvate ratio should remain 2:1 to ensure ROS detoxification.

### Quantitative physiological analysis of growth parameters

For the following analysis only steady states below *D* = 0.050 h^−1^ were considered, as we expect the results at and beyond this *D* to be heavily influenced by biofilm formation. Data points at *D* = 0.0124 h^−1^ and 0.0146 h^−1^ are from three different bioreactors that were used for lab scale biomass production (data not shown).

**Figure 6:**
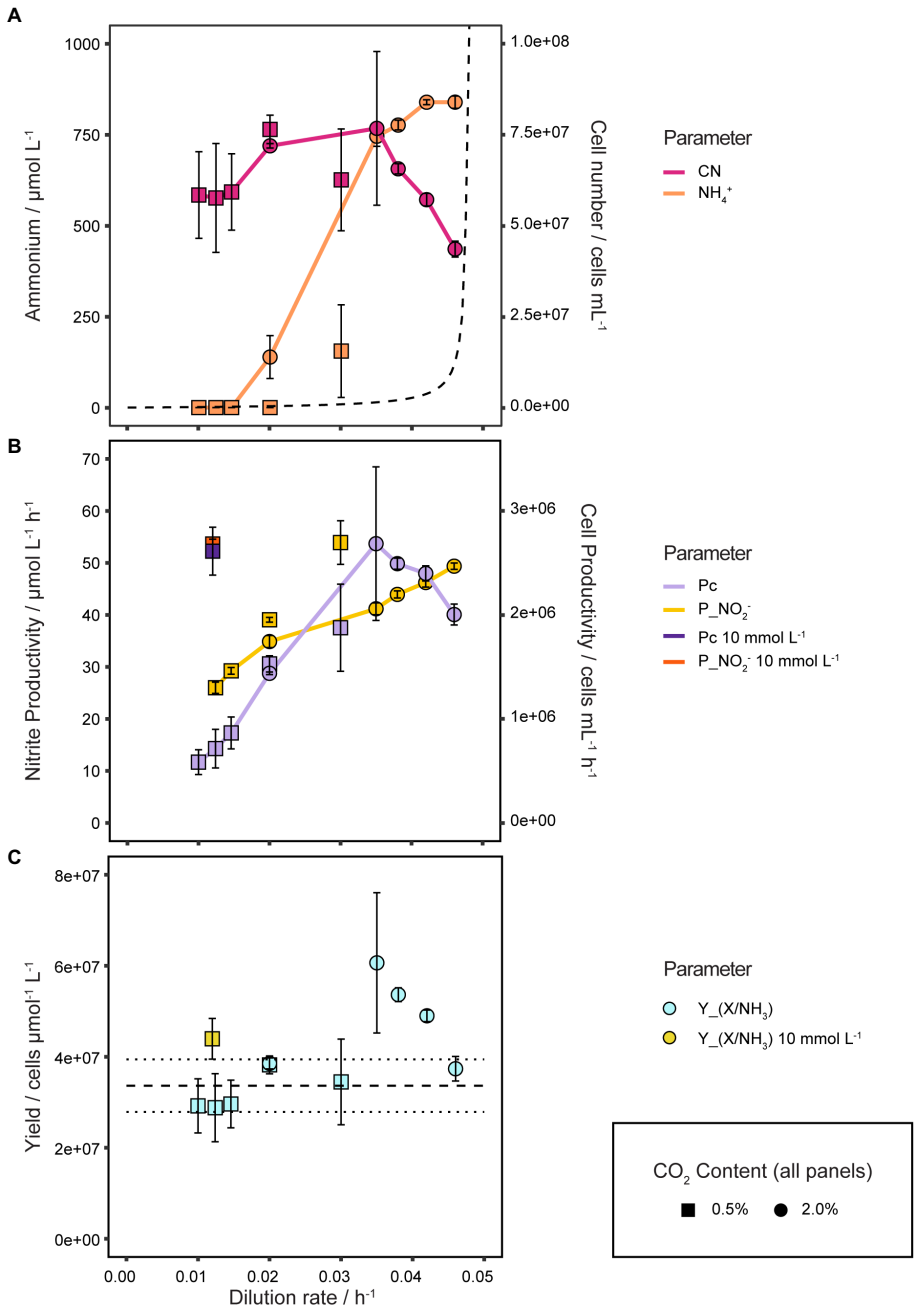
Quantitative analysis of physiological growth parameters. **(A)** Cell number and *S* in response to different *D* and CO_2_ concentrations. The dashed line shows the theoretical development of *S* with increasing *D* assuming Monod kinetics, *Ks* equal to *K*_*m*(app)_ of 5.4 μmol L^−1^ and μ_max_ of 0.048 h^−1^, illustrating the difference to the observed growth behavior of *N. viennensis*. NH_4_^+^ and cell number curves show the mean values of biological duplicates, of which each NH_4_^+^ value consists of technical triplicates (samples taken at different time points), and error bars represent the standard deviation of the mean. **(B)** Nitrite (P_NO2_) and cell productivity (P_C_) of 2 and 10 mmol L^−1^ NH_4_Cl continuous cultures at different *D* and CO_2_ concentrations. P_NO2_ and P_C_ neither correlate in a fixed 1:1 ratio as would be expected for 0.5% nor 2% CO_2_ gassed cultures, indicating a variable Y_(X/NH3)_. P_NO2_ and P_C_ points show mean values of biological duplicates, triplicates for *D* of 0.0124 h^−1^ and 0.0146 h^−1^, and error bars represent the standard deviation of the mean. **(C)** Y_(X/NH3)_ in response to different *D* and CO_2_ concentrations. Y_(X/NH3)_ points show mean values of biological duplicates, triplicates for *D* = 0.0124 h^−1^ and 0.0146 h^−1^, and error bars represent the standard deviation of the mean. Points with small standard deviation may not have visible error bars.

As shown in Figure 6A the cell number of *N. viennensis* increased slightly with *D* from 5.84·10^7^ ± 1.19·10^7^ cells mL^−1^ at *D* = 0.010 h^−1^ up to 7.67·10^7^ ± 2.11·10^7^ cells mL^−1^ at *D* = 0.035 h^−1^ from where it decreased linearly with *D* to 4.36·10^7^ ± 2.18·10^6^ cells mL^−1^ at *D* = 0.046 h^−1^.

NH_4_^+^ was completely consumed up to *D* = 0.020 h^−1^ by cultures gassed with 0.5 Vol.-% CO_2_. For 2 Vol.-% CO_2_ gassed cultures *S* started to accumulate at *D* = 0.020 h^−1^ to 138 ± 58.9 μmol L^−1^ and increased steeply up to 745.5 ± 27.6 μmol L^−1^ NH_4_^+^ at *D* = 0.035 h^−1^ where it began to stagnate and reach a maximum of 850.0 ± 11.6 μmol L^−1^ at *D* = 0.046 h^−1^. Despite the early onset of increasing *S*, the cell concentration remained surprisingly stable up to *D* = 0.035 h^−1^. Conversely, while the cell number was decreasing with *D* from 0.035 to 0.046 h^−1^, *S* increased only marginally, thus changes in *S* appeared to be not reflected in the cell number and vice versa.

NO_2_^−^ and cell productivities (P_NO2_, P_C_) are shown in Figure 6B. P_NO2_ increased logarithmically from *D* of 0.010 to 0.020 h^−1^ and from there on in a linear way up to 49.6 ± 0.3 μmol L^−1^ h^−1^ at *D* = 0.046 h^−1^. P_C_ increases linearly from 5.84·10^5^ ± 1.19·10^5^ cells mL^−1^ h^−1^ at *D* = 0.010 h^−1^ up to 2.69·10^6^ ± 7.39·10^5^ cells mL^−1^ h^−1^ at *D* = 0.035 h^−1^ from which point it started to decline to 2.00·10^6^ ± 1.00·10^5^ cells mL^−1^ h^−1^ at *D* = 0.046 h^−1^. As a result of a variable Y_(X/NH3)_, P_NO2_ and P_C_ do not correlate in a fixed 1:1 ratio as would be expected. For comparison P_NO2_ and P_C_ of 10 mmol L^−1^ cultures at *D* = 0.012 h^−1^ are also shown in Figure 6C with 53.6 ± 0.9 μmol L^−1^ h^−1^ and 2.61·10^6^ ± 2.30·10^5^ cells mL^−1^ h^−1^, respectively. The highest P_C_ was reached at *D* = 0.035 h^−1^ which is therefore the optimal *D* for biomass production for *N. viennensis*.

As shown in Figure 6C an average Y_(X/NH3)_ of 3.36·10^7^ ± 5.77·10^6^ cells μmol^−1^ L^−1^ was determined from the batch cultures before changing into continuous mode. Y_(X/NH3)_ from steady states at *D* of 0.010 to 0.030 h^−1^ or 10 mmol L^−1^ NH_4_Cl cultures (*D* from 0.005 to 0.012 h^−1^) were well within this range, but from *D* = 0.035 h^−1^, Y_(X/NH3)_ were highly elevated with 6.12·10^7^ ± 1.68·10^7^ cells μmol^−1^ L^−1^ and decreased to 3.79·10^7^ ± 1.90·10^6^ cells μmol^−1^ L^−1^ at *D* = 0.046 h^−1^. Cultures at *D* of 0.020 h^−1^ gassed with 0.5 Vol.-% and 2 Vol.-% CO_2_ had very much the same Y_(X/NH3)_ with 3.82·10^7^ ± 1.96·10^6^ cells μmol^−1^ L^−1^ and 3.86·10^7^ ± 3.34·10^5^ cells μmol^−1^ L^−1^, respectively. While Y_(X/NH3)_ of steady states at low *D* are comparable to batch cultures, at higher *D*, Y_(X/NH3)_ increased up to 81.7% (*D* = 0.035 h^−1^) compared to batch cultures.

## Discussion

The substrate affinity, together with μ, are very important parameters for understanding the ecological strategy of an organism (*r*- and *k*-strategists). Due to their very high substrate affinity and low μ, AOA are regarded as typical k-strategists but the growth behavior of *N. viennensis* observed in this study can not sufficiently be described with Monod kinetics or more sophisticated models like the Briggs-Haldane model. The strong increase of *S* at *D* = 0.035 h^−1^ would indicate that *D* was already close to μ_max_, but then *S* should have increased right after the start of the continuous culture and further increases of *D* should have resulted in even stronger increases of *S*. Instead *S* plateaued around 800 μmol L^−1^ NH_4_^+^ while the cell number decreased with *D* increasing up to 0.046 h^−1^. An explanation for this behavior would be the formation of a biofilm that would retain cells in the reactor, but at the same time decrease planktonic cell concentrations. Thus, the activity of the whole system would increase while at the same time the measured cell concentrations would decrease, which describes the results. The stable *S* concentrations and sposntaneous but isochronal activity increases are difficult to integrate into this explanation. Only very strong increases of *D* like from 0.050 h^−1^ to 0.060 h^−1^ and further to 0.070 h^−1^ caused *S* to increase temporarily while cell concentrations remained surprisingly stable – considering a μ_max_ of 0.048 h^−1^ for *N. viennensis*. At *D* > μ_max_ cells should usually be washed out over time, but due to the seeding of cells by the biofilm this phenomenon was not observed. The linear decrease of *S* at *D* = 0.060 h^−1^ might be the result of an increase in active biofilm biomass, that reaches its limits at *D* = 0.070 h^−1^. This would explain the very stable concentrations of *S* after the spontaneous activity increase. These increases of activity might be induced by quorum sensing and be dependent on cell density in the biofilm, which should gradually increase with time until a maximum is reached. This would provide a robust principle, which could explain the synchronistic nature of this very unusual physiological phenomenon. The mechanistic principle of how the cells are able to increase their substrate affinity abruptly could be explained by the expression of multiple *amo*C genes, of which the genome of *N. viennensis* contains six, which is likely to be the subunit that contains the catalytic center of the AMO protein complex. This assumption is based on recent findings of a bacterial particulate methane monooxygenase (Ross et al., 2019).

In addition, there seems to be another regulatory element in the growth behavior of *N. viennensis* that was observed with 2 mmol L^−1^ and 10 mmol L^−1^ NH_4_^+^ continuous cultures. At a certain *D* the organism consumes NH_3_ at a slightly lower rate than provided, thus slowly increasing *S* until it finally stabilizes at roughly 50% of *S_i_*. Once stabilized, *S* only marginally increases with *D* unless very strong changes are induced. This phenomenon appears to be linked to an increase in Y_(X/NH3)_, which seems to be highly elevated at *D* > 0.030 h^−1^. However, we must state that we analyze the biomass productivity in relation to the energy metabolism, assuming that energy is limiting the biomass formation and not carbon, which was provided in excess to *N. viennensis* during chemostat and biofilm formation experiments.

In a steady state Y_(X/NH3)_ might be increased because the enzymatic machinery and metabolic networks can be fine tuned to a stable environmental condition. A batch culture needs to adapt constantly to the changing environment which requires energy. However, this phenomenon occurs in every organism and can not describe the vast increase of Y_(X/NH3)_ in continuous cultures of up to 81.7% compared to batch cultures. This might only be explained by taking into account the unique nature of archaeal ammonia oxidation, which produces significant amounts of ROS that can destroy the cells if not taken care of by the environment (e.g., by catalases, alpha keto-acids). Thus, there is a strong selection pressure on the organism to regulate its activity in accordance to its environment. Given the wide distribution of AOA, it seems that these organisms might have evolved elaborate metabolic regulations to enable them to thrive. The production of ROS by AOA might thus be key to understanding their ecological success, because it could also be used by the organisms to generate substrates from the environment by oxidative decarboxylation (Kim et al., 2016) or oxidative deamination (Akagawa et al., 2002) of organic matter. This would supply the organism with both CO_2_ and NH_3_ and could explain why some AOA, like the members of the Nitrosocosmicus clade, are highly abundant in very organic rich soils. However, the conditions in soil, the primary habitat of *Nitrososphaera* spp., are not close to the conditions inside a bioreactor that operates in continuous culture mode. Hence, biofilm formation might be a preferred form of life for soil AOA, or could confer some resistance to selection *via* μ. In addition, this kind of metabolism, however, requires a high degree of metabolic regulation which might be enabled by the transcription apparatus of archaea, which is usually summarized as a simplified version of the eukaryotic machinery, even though this observed simplicity is under constant revision as new insights are accumulated (Gehring et al., 2016). The unique nature of archaea and their gene regulation could therefore be responsible for this form of NH_3_ oxidation. It appears that the guiding principle behind the growth dynamics of *N. viennensis*, and probably other AOA, is not substrate affinity but the maximization of Y_(X/NH3)_ and therefore optimal utilization of a usually very limited substrate.

These insights into the regulation of the energy metabolism of *N. viennensis* have important implications for further bioprocess development to optimize biomass productivity. Due to the increase of Y_(X/NH3)_ with higher *D*, biomass productivity also increases despite high *S* concentrations. CO_2_ concentration in the in-gas plays a crucial role in the way that it does not effect Y_(X/NH3)_ but μ_max_ and *S*. An optimal CO_2_ concentration should not reduce μ_max_ but at the same time minimize *S* and therefore maximize the cell concentration at a given *D* and *S*_i_. For higher concentrated feed medium it is probably worth in terms of biomass productivity to accept higher *S* concentrations as long as *D* positively affects Y_(X/NH3)_ and biomass productivity. It is important to note that the NH_4_Cl to pyruvate ratio should always be at least in a 2:1 to prevent self toxification by endogenously produced ROS. Depending on the scientific question, higher NH_4_^+^ concentrations might not be desirable as it certainty influences biomass composition and gene expression. For compounds of interest that cannot be recombinantly produced, higher substrate concentrations might very well be the method of choice as it looks promising to obtain good biomass productivities if further improved.

For the production of biomass, biofilm formation is not favorable as it reduces the amount of cells that can be harvested and thus the effective Y_(X/NH3)_. Elevated temperatures are known to increase biofilm formation (Qureshi et al., 2005), therefore lowering the temperature might be a solution but at the cost of reducing μ_max_. Reducing the CO_2_ concentration will also likely reduce biofilm formation, because carbonate precipitate will be reduced and therefore also available surface that can induce biofilm formation. On the other hand, to understand the ecological function and behavior of *N. viennensis*, it would be very important to study the organism in biofilms, as this is more likely to be its prevalent form in soils. Gene abundances based on 16S rRNA or *amo*A are often used to infer NH_3_ oxidizing activity of AOA in soils, but discrepancies with activity measurements of soil incubations are known. Estimates from this study show that only 14.67% of the cells in the biofilm would have been active at their maximum capacity. These results were obtained from two calculations with different assumptions in two different reactors in two different experiments. However, independent of the discrepancy of the results, this high ratio of inactive cells could also explain earlier findings of inactive cells that started the hypothesis of mixotrophic AOA together with the growth enhancing effect of alpha keto acids (Mußmann et al., 2011; Tourna et al., 2011). As biofilms have the potential for very complex cell interactions, it might also be that “inactive” cells simply perform different tasks in the biofilm beside NH_3_ oxidation. Regardless, careful validation and/or correction of these results with future experiments would be needed to have a more accurate picture of the behavior of AOA biofilms.

## Data availability statement

The raw data supporting the conclusions of this article will be made available by the authors, without undue reservation.

## Author contributions

MM, CS, and SK-MRR: conceived and designed study. MM, MAM, and SK-MRR: performed research. MM and LH: analyzed the data. MM, LH, and SK-MRR: contributed new methods or models and wrote the paper. All authors read and approved the manuscript.

## Funding

Open access funding by the University of Vienna.

## Conflict of interest

SK-MRR was employed by the company Arkeon GmbH.

The remaining authors declare that the research was conducted in the absence of any commercial or financial relationships that could be construed as a potential conflict of interest.

## Publisher’s note

All claims expressed in this article are solely those of the authors and do not necessarily represent those of their affiliated organizations, or those of the publisher, the editors and the reviewers. Any product that may be evaluated in this article, or claim that may be made by its manufacturer, is not guaranteed or endorsed by the publisher.

## Supplementary material

The Supplementary material for this article can be found online at: https://www.frontiersin.org/articles/10.3389/fmicb.2023.1076342/full#supplementary-material
