# Supplementary material for: Analysis of biomass productivity and physiology of Nitrososphaera viennensis grown in continuous culture
Source: Front Microbiol. 2023 Feb 16;14:1076342. doi: 10.3389/fmicb.2023.1076342 (PMC9978112; doi:10.3389/fmicb.2023.1076342)
Supplement: Supplementary file 1 [file Data_Sheet_1.docx]

Supplementary Material

# Supplementary Figures and Tables

## Supplementary Figures

**Supplementary Figure 1.** Effect of gassing on batch cultures. NH_4_^+^, NO_2_^-^ and dO_2_ concentrations of open batch cultures gassed with 2 sL L^-1^ h^-1^ air (gas) or closed batch cultures (w/o gas) all containing 2 mmol L^-1^ NaHCO_3_ as C-source. Cultures gassed with air had decreased µ of 0.0276 ±0.0001 h^-1^ compared to closed batch cultures with µ 0.0445 ±0.0004 h^-1^. NH_4_^+^, NO_2_^-^ and dO2 curves show mean values of biological duplicates and error bars represent the standard deviation of the mean.

**S****upplementary Figure 2.** Effect of gassing rate and in-gas flow composition on batch cultures. NH_4_^+^, NO_2_^-^ and dO_2_ concentrations of open batch cultures gassed with 1 sL L^-1^ h^-1^ air (21% O_2_) or air/N_2_ mix (12.6% O_2_) to reduce the dO_2_ concentration. NO_2_^-^ curves of closed batch (w/o gas) and open batch cultures gassed with 2 sL L^-1^ h^-1^ air (gas) are shown as comparison. Reducing the gassing rate did only marginally effect µ (0.0268 ±0.0001 h^-1^) while decreasing dO_2_ concentration did decrease µ to 0.0206 ±0.0001 h^-1^. The increase of dO_2_ at 135 h was due to technical issues. NH_4_^+^, NO_2_^-^ and dO_2_ curves show mean values of biological duplicates and error bars represent the standard deviation of the mean.

**Supplementary Figure 3.** Maximum specific growth rate (µ_max_) of *N. viennensis*. µ of closed batch cultures grown at different dO_2_ concentrations in serum flasks with 0.5% CO_2_ in the atmosphere. Highest µ of 0.0508 ±0.0005 h^-1^ was achieved at 53.5 µmol L^-1^ dO_2_ (5% O_2_ in the gas phase) compared to 0.0484 ±0.0004 h^-1^ at 224.7 µmol L^-1^ (21% O_2_ in the gas phase). Due to the remarkable little effect of dO_2_ on µ, 21% O_2_ were used for continuous cultures and a µ_max_ of 0.0484 h^-1^ was therefore assumed. µ values show the mean of quadruplets and error bars represent the standard deviation of the mean.

**Supplementary Figure 4.** Increase of cell number based growth rate in continuous cultures. µ of BR1 and BR2 based on cell concentration from D 0.035 h^-1^ to 0.060 h^-1^. Even though the cell concentration decreased from D 0.035 h^-1^ to D 0.050 h^-1^, µ increased with D but remained slightly below the set value until D 0.050 h^-1^ where it started to surpass D leading to an increase in cell concentration.

**Supplementary Figure 5.** Boxplots of NH_4_^+^ and cell number of continuous cultures at high dilution rates. (A) NH_4_^+^ concentrations of sections A (382 to 990 h), B (1032 to 1654 h), C (1796 to 2035 h) and D (2062 to 2402 h). Different steady states of BR1 and BR2 in sections C and D are signified by the corresponding number. Outliers known to be caused by technical issues (1271, 1295, 1343 and 1346 h) were removed from the dataset. (B) Cell number of BR1 and BR2 of sections A to D, the different steady states of BR1 and BR2 in sections C and D are signified by the corresponding number.

**Supplementary Figure 6.** Inhibitory effect of nitrite on the growth rate. 20 mL batch cultures grown in 30 mL polystyrene tubes with 1 mmol L^-1^ NH_4_^+^ and 0 to 19 mmol L^-1^ NO_2_^-^ starting concentrations. NO_2_^-^ exhibited only a weak linear inhibitory effect on µ with 0.0342 ±0.0004 h^-1^ to 0.0411 ±0.0005 h^-1^ for 19 and 0 mmol L^-1^ NO_2_^-^ starting concentrations respectively. Initial pH of 7.5. µ values show the mean of triplicates and error bars represent the standard deviation of the mean.
